# Supplementary material for: Control of artificial membrane fusion in physiological ionic solutions beyond the limits of electroformation
Source: Nat Commun. 2024 May 28;15:4524. doi: 10.1038/s41467-024-48875-0 (PMC11133453; doi:10.1038/s41467-024-48875-0)
Supplement: Supplementary file 1 — Supplementary Information [file 41467_2024_48875_MOESM1_ESM.pdf]

[Supplementary information]

Control of artificial membrane fusion in physiological ionic solutions beyond the limits of electroformation

Bong Kyu Kim[1,2,†], Dong-Hyun Kang[3,†], Junhyuk Woo[1], Wooseung Yoon[1], Hyunil Ryu[1], Kyungreem Han[1], Seok Chung[2], Tae Song Kim[1,*]

[1] Convergence Research Center for Brain Science, Korea Institute of Science and Technology, 5, Hwarang-ro 14-gil, Seongbuk-gu, Seoul 02792, Republic of Korea

[2] Department of Mechanical Engineering, Korea University, 145 Anam-ro, Seongbuk-gu, Seoul, 02841, Republic of Korea

[3] Bionics Research Center, Korea Institute of Science and Technology, 5, Hwarang-ro 14-gil, Seongbuk-gu, Seoul 02792, Republic of Korea

† These authors contributed equally to this work: Bong Kyu Kim, Dong-Hyun Kang

* Corresponding author: tskim@kist.re.kr

[Supplementary Methods]

Mechanical analysis of 3-dimensional freestanding lipid bilayer

Here we provide a mechanical analysis of 3D freestanding lipid bilayers (3DFLBs) using the mathematically intensive geometric theory of spherical membranes[1–3]. Primary purposes include 1) generation of 3D geometries of 3DFLBs and 2) characterization of mechanical changes of 3DFLBs upon applied hydraulic pressure, based on the biophysics of 1,2-dioleoyl-sn-glycero-3-phosphocholine (DOPC) bilayers[4–7]. Three DOPC-3DFLBs systems, each representing typical topologies during 3DFLBs formations, are examined — the case where a small vesicle is laid on the bottom of the microwell without contact with the sidewall of the microwell (*boundary condition 1*; *BC-1*), in which a large vesicle is restricted due to a bilayer/side wall border (*BC-2*), and the situation where an outward vesicle is placed on the top of the microwell along its circular edge (*BC-3*). All the systems are analyzed under a wide range of applied pressures.

1. Spherical surface geometry of 3D freestanding lipid bilayer structure

We first consider a clamped 3DFLBs surface geometry based on the standard spherical shell theory[1,2]. As shown in Supplementary Figure 16, an infinitesimal volume element of the clamped surface (*OABC*) is defined by two meridional sections for azimuthal angles ψ and $\psi + d\psi$ and two conical surfaces for the polar angles θ and $\theta + d\theta$. The thickness of the volume element is assumed constant, denoted by h. Then, the volume elements are integrated over the whole 3DFLBs spherical surface.

Three vector components, u, v, and w at the vertex O, are defined whereby u and v denote tangential displacements along the x- and y-axis, respectively, and w means the displacement in the direction normal to the spherical surface. The stresses parallel to the

surface (in the unit of N/m^2), σ_x (negative direction of x-axis) (Eq. 15), and σ_y (for y-axis) (Eq. 16) are defined (Supplementary Fig. 16). Corresponding forces (per unit length) along the x- and y-axis are written in the forms of $N_x = \int_{-h/2}^{+h/2} \sigma_x dz$ (Eq. 17) and $N_y = \int_{-h/2}^{+h/2} \sigma_y dz$ (Eq. 18). The bending moment (per unit length) parallel to the x-axis reads $M_x = \int_{-h/2}^{+h/2} z\sigma_y dz$ (Eq. 19) and that parallel to the y-axis is $M_y = \int_{-h/2}^{+h/2} z\sigma_x dz$ (Eq. 20).

2. *Characterization of mechanical changes of 3DFLBs with hydraulic pressure*

To capture the mechanical changes of 3DFLBs upon applying external hydraulic pressure, we analyze the primary mechanical descriptors (Supplementary Fig. 18) for the 3DFLBs surface geometry (Supplementary Fig. 16) using the 3DFLBs surface model. We conduct simulations for the three typical cases during 3DFLBs formations (Supplementary Fig. 17) using realistic biophysical parameters for DOPC bilayers[4–6].

2.1. *Dynamics of 3DFLBs systems*

The dynamics of the relations of the mechanical descriptors, as depicted in Supplementary Figure 18, are described by six coupled differential equations (Eqs. (1)–(6)), which are solved using a damped Newton's method with an affine-invariant criterion function[8] and a 4th-order collocation algorithm[9] via SciPy Python library (version 1.10.0)[10].

Simulations are run under three mechanical boundary conditions: (*BC-1*) a small vesicle is laid on the bottom of the microwell, not interacting with the side wall of the microwell; (*BC-2*) a large vesicle is in contact with the microwell at the bilayer/sidewall interface; (*BC-3*) an outward vesicle is placed on the top of the microwell along its circular edge (Supplementary Fig. 17). In all cases, we set $v = 0$, both u and w depend on θ but are independent of ψ, assuming azimuthally symmetric deformations of 3DFLBs upon applying hydraulic pressure.

The parameters are adopted from biophysical experiments on DOPC bilayers: the value of Young's modulus (E = 1.3×10^4 kPa) is taken from the atomic force microscopy (AFM) experiment on the DOPC bilayer[4], Poisson ratios (v) of the DOPC bilayer are in the range of 0.450–0.501[5], and the bilayer thickness is set to 40 Å according to the measurement on DOPC-GUV (giant unilamellar vesicle)[6]. A complete list of parameters is presented in Supplementary Table 1.

$$\frac{\partial N_x}{\partial \theta} = -\left(N_x - N_y\right)\cot\theta + Q_x, \tag{1}$$

$$\frac{\partial Q_x}{\partial \theta} = -Q_x\cot\theta - \left(N_x + N_y\right) - qa, \tag{2}$$

$$\frac{\partial M_y}{\partial \theta} = -\left(M_y - M_x\right)\cot\theta + Q_x a, \tag{3}$$

$$\frac{\partial u}{\partial \theta} = w + a\varepsilon_x^{(0)}, \tag{4}$$

$$\frac{\partial w}{\partial \theta} = \widetilde{w}, \tag{5}$$

$$\frac{\partial \widetilde{w}}{\partial \theta} = a^2\chi_x - \left(w + a\varepsilon_x^{(0)}\right). \tag{6}$$

Here, Q_x denotes a vertical shearing force acting on the face normal to the x-axis[1,2], a means radius of curvature, q is the additional hydraulic pressure on 3DFLBs (we assume that the external hydraulic pressure Δp acts as the additional hydraulic pressure without losses, i.e., $q = \Delta p$) added to the default pressure in the microfluidic channel (p_0), $\varepsilon_x^{(0)}$ the unit elongation of the middle surface along the x-axis (Eq. 7), and χ_x the change in the principal curvature on the xz-plane (Eq. 9).

2.2. Relations of mechanical descriptors

We write the unit elongation of the middle surface along the x- and the y-axis,

respectively, in the form:

$$\varepsilon_x^{(0)} = \frac{du}{a d\theta} - \frac{w}{a}, \tag{7}$$

$$\varepsilon_y^{(0)} = \frac{u \cos\theta}{a \sin\theta} - \frac{w}{a}, \tag{8}$$

and changes in curvatures are governed by the following equation:

$$\chi_x = \frac{1}{r_x'} - \frac{1}{r_x} = \frac{d^2 w}{a^2 d\theta^2} + \frac{du}{a^2 d\theta}, \tag{9}$$

$$\chi_y = \frac{1}{r_y'} - \frac{1}{r_y} = \left(\frac{u}{a^2} + \frac{dw}{a^2 d\theta}\right) \cot\theta, \tag{10}$$

where r_x and r_y denote the radius of principal curvature on the *xz*- and *yz*-plane, respectively, and r_x' and r_y' those upon mechanical changes.

Unit elongations along the *x*-axis of a thin lamina and that for the *y*-axis at a distance *z* from the middle surface take the forms:

$$\varepsilon_x = \frac{\varepsilon_x^{(0)}}{1 - z/r_x} - \frac{z}{1 - z/r_x}\left[\frac{1}{\left(1 - \varepsilon_x^{(0)}\right) r_x'} - \frac{1}{r_x}\right], \tag{11}$$

$$\varepsilon_y = \frac{\varepsilon_y^{(0)}}{1 - z/r_y} - \frac{z}{1 - z/r_y}\left[\frac{1}{\left(1 - \varepsilon_y^{(0)}\right) r_y'} - \frac{1}{r_y}\right]. \tag{12}$$

Assuming that the bilayer thickness *h* of the shell is much smaller than the radius of curvature (i.e., z/r_x and z/r_y are negligible) and ignoring the effect of elongations $\varepsilon_x^{(0)}$ and $\varepsilon_y^{(0)}$ on the curvature, Eqs. (11) and (12) are reduced as below.

$$\varepsilon_x = \varepsilon_x^{(0)} - z\left(\frac{1}{r_x'} - \frac{1}{r_x}\right) = \varepsilon_x^{(0)} - \chi_x z, \tag{13}$$

$$\varepsilon_y = \varepsilon_y^{(0)} - z\left(\frac{1}{r_y'} - \frac{1}{r_y}\right) = \varepsilon_y^{(0)} - \chi_y z. \tag{14}$$

The stresses parallel to the surface (in the unit of N/m^2), σ_x (negative direction of *x*-

axis) and σ_y (for y-axis) read:

$$\sigma_x = \frac{E}{1-\nu^2}\left[\varepsilon_x^{(0)} + \nu\varepsilon_y^{(0)} - z(\chi_x + \nu\chi_y)\right], \tag{15}$$

$$\sigma_y = \frac{E}{1-\nu^2}\left[\varepsilon_y^{(0)} + \nu\varepsilon_x^{(0)} - z(\chi_y + \nu\chi_x)\right], \tag{16}$$

where E denotes Young's modulus and v means Poisson ratio.

The forces per unit length along the x- and y-axis take the forms:

$$N_x = \int_{-\frac{h}{2}}^{+\frac{h}{2}} \sigma_x dz = \frac{Eh}{1-\nu^2}\left(\varepsilon_x^{(0)} + \nu\varepsilon_y^{(0)}\right), \tag{17}$$

$$N_y = \int_{-\frac{h}{2}}^{+\frac{h}{2}} \sigma_y dz = \frac{Eh}{1-\nu^2}\left(\varepsilon_y^{(0)} + \nu\varepsilon_x^{(0)}\right). \tag{18}$$

The bending moment per unit length parallel to the x- and that for y-axis are defined as follows:

$$M_x = \int_{-\frac{h}{2}}^{+\frac{h}{2}} z\sigma_y dz = -D(\chi_y + \nu\chi_x), \tag{19}$$

$$M_y = \int_{-\frac{h}{2}}^{+\frac{h}{2}} z\sigma_x dz = -D(\chi_x + \nu\chi_y). \tag{20}$$

The flexural rigidity (D) as a function of applied pressure is written in the form:

$$D = \kappa\left(b\frac{(q+p_0)^\delta}{(q+p_0)^\delta + c^\delta} + d\right)\frac{Eh^3}{12(1-\nu^2)}, \tag{21}$$

where δ is the exponent of the pressure dependence of D, b, c, and d are the constants for the pressure dependence, p_0 denotes the default pressure of the system, q is the additional pressure, and κ represents the contribution from other deformation factors (e.g., electric field and ionic strength) in addition to pressure. This equation agrees well with the experimental result on DOPC-GUV that the flexural rigidity of the DOPC bilayer nonlinearly increases against a wide range of applied pressure[7].

The dynamic equations (Eqs. (1)-(6)) are obtained considering that all the individual applied forces with respect to the x-, y-, and z-axis are equilibrated and assuming the azimuthally symmetric deformation of 3DFLBs. These are expressed in Eqs. (22)-(24): the forces along the x-axis (Eq. 22), that for the z-axis (Eq. 23), and the moments of forces to the y-axis (Eq. 24).

$$\frac{dN_x}{d\theta} + \left(N_x - N_y\right)\cot\theta - Q_x = 0 \tag{22}$$

$$\frac{dQ_x}{d\theta} + Q_x \cot\theta + N_x + N_y + qa = 0 \tag{23}$$

$$\frac{dM_y}{d\theta} + \left(M_y - M_x\right)\cot\theta - Q_x a = 0 \tag{24}$$

2.3. Mechanical changes of 3DFLBs

The mechanical changes of 3DFLBs are depicted in Supplementary Figure 19-24.

[Supplementary Figures]

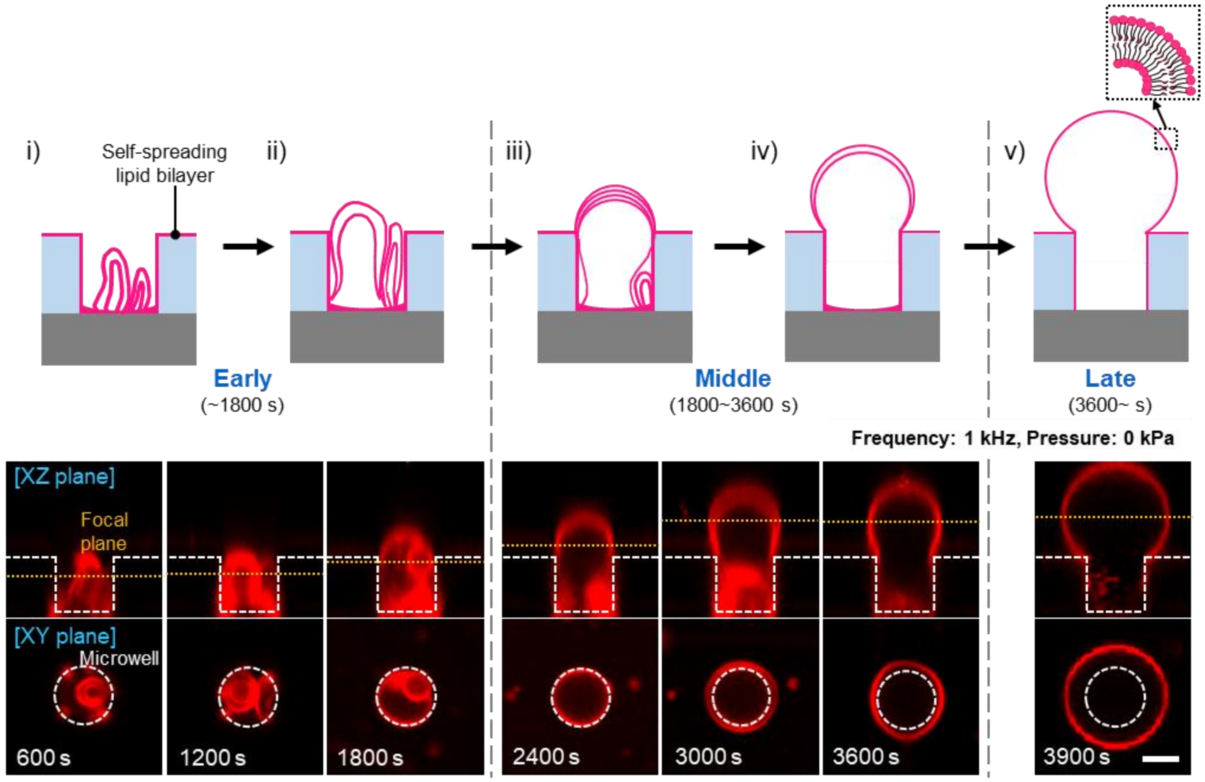

Supplementary Figure 1. Schematics and representative cross-sectional confocal microscopy images of the whole 3DFLBs generation period using pristine electroformation without hydraulic pressure at an AC frequency of 1 kHz: i-ii) multilayer lipid membranes explosively swelled within 600 s by encountering solution and then growing gradually, fused each other within 1800 s in the early stage; iii-iv) development of the multilayer membranes and fusion with other membranes in the middle stage; and v) final 3DFLBs, formed by the fusion of multilayer freestanding lipid membranes in the late stage. It is clear that the confined space of microwells, particularly the peripheral sidewalls of individual microwells, promotes the fusion process by improving the proximity of controlled random swells and concentrating additional forces between the contact areas due to the expansion of membrane swells in microwells during electroformation. Scale bar: 5 μm. Lipid composition: DOPC with 1 mol% Rhod-PE.

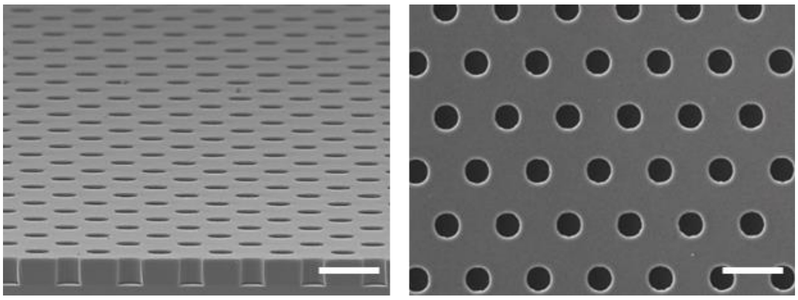

Supplementary Figure 2. SEM images of the SU-8 microwell array substrate. The diameter of the microwell is 8 μm, the thickness of the SU-8 is 8 μm, and the pitch of each microwell is 20 μm. The SU-8 microwell array is designed and fabricated with a high density of ~ 300,000 #/cm^2. Scale bar: 20 μm.

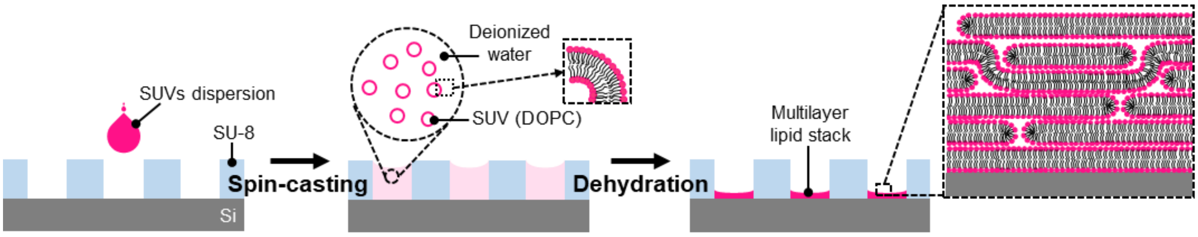

Supplementary Figure 3. Schematic illustration of lipid patterning into the microwells. Dropped SUVs dispersion is isolated and patterned into the microwells via spin-coating. The lipid solution is dehydrated, resulting in multilayer lipid stacks inside the microwell.

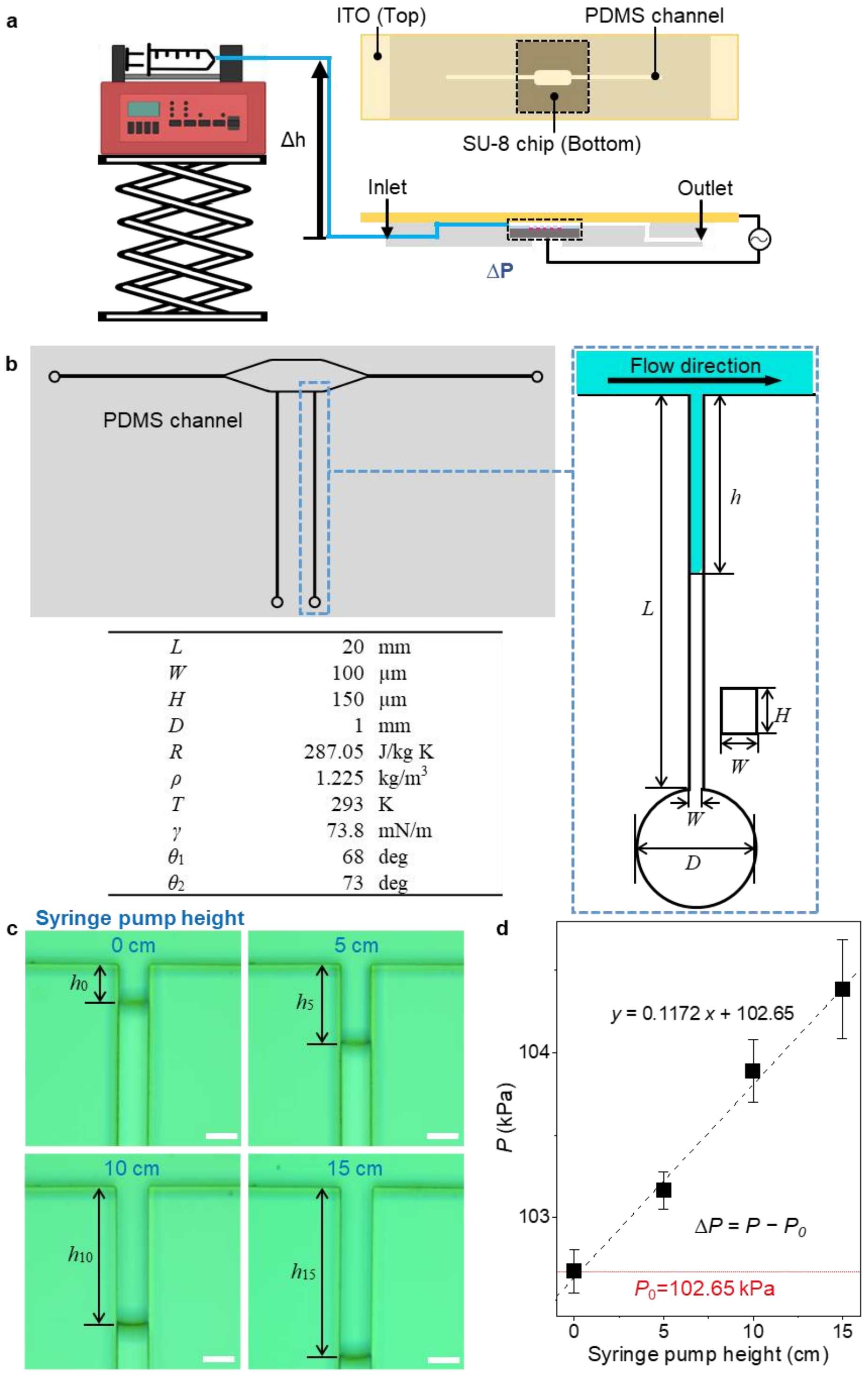

Supplementary Figure 4. Precise control and evaluation of hydraulic pressure applied in the microchannel based on the height of the syringe pump. (a) Hydrostatic pressure of the syringe, which is affected by the height of the syringe pump elevation in reference to the location of the microchannel, producing the hydraulic pressure in the microchannel. (b) Dimensions of the

microchannel with two pressure taps and parameters for calculating the pressure in the microchannel. (c) Microscope images of the measured *h* (displacement of the meniscus in the pressure tap) at various heights of the syringe pump. Scale bar: 100 μm. (d) Calculated microchannel pressure based on the measured *h* values according to the heights of the syringe pump. Here, P_0 = 102.65 kPa, which is the reference pressure, meaning that the height of the syringe pump elevation is zero. The microfluidic channel has flow resistance, so the initial pressure is P_0. In this study, the hydraulic pressure (applied pressure) was calculated as $\Delta P = P - P_0$.

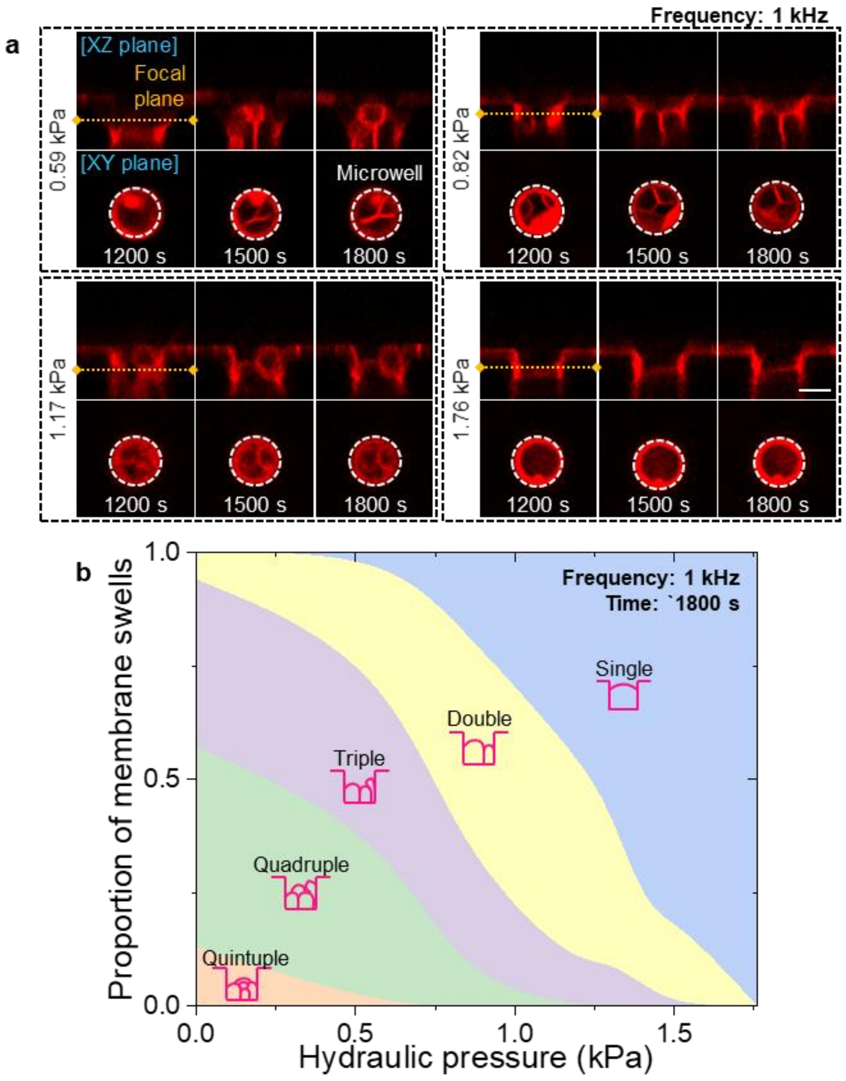

Supplementary Figure 5. (a) Cross-sectional confocal fluorescence microscopy images of membrane swells in the early stage of the 3DFLBs fabrication process at an AC frequency of 1 kHz and different hydraulic pressures. Scale bar: 5 μm. (b) Number proportion of membrane swells as a function of hydraulic pressure at the end of the early stage (1800 s), where the 3DFLBs were produced at an AC frequency of 1 kHz.

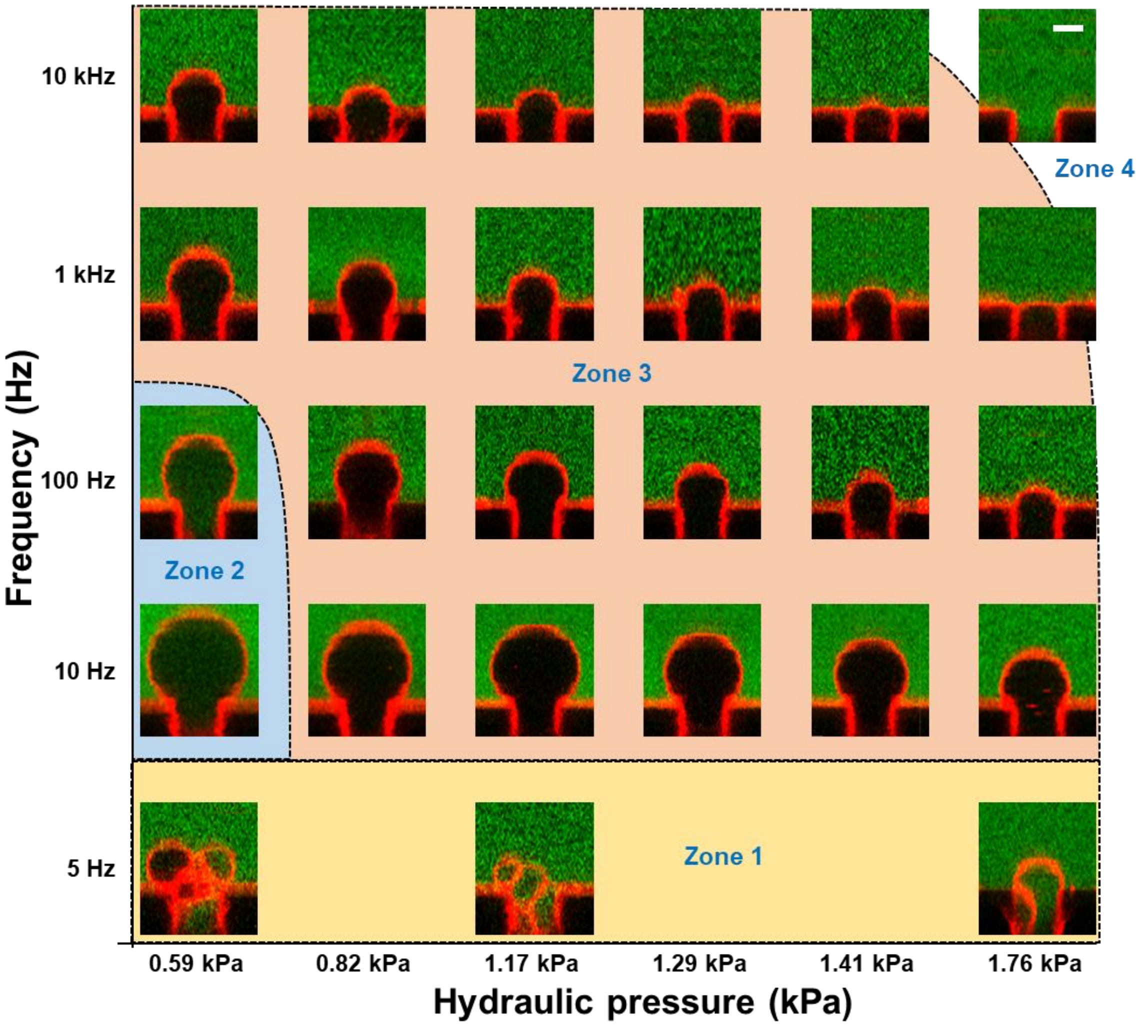

Supplementary Figure 6. Tunable shape and sealing characteristics of 3DFLBs fabricated in sucrose solution at various AC frequencies and hydraulic pressures. The cross-sectional confocal fluorescence microscopy images of the 3DFLBs were captured at different AC frequencies and hydraulic pressures. Scale bar: 5 μm.

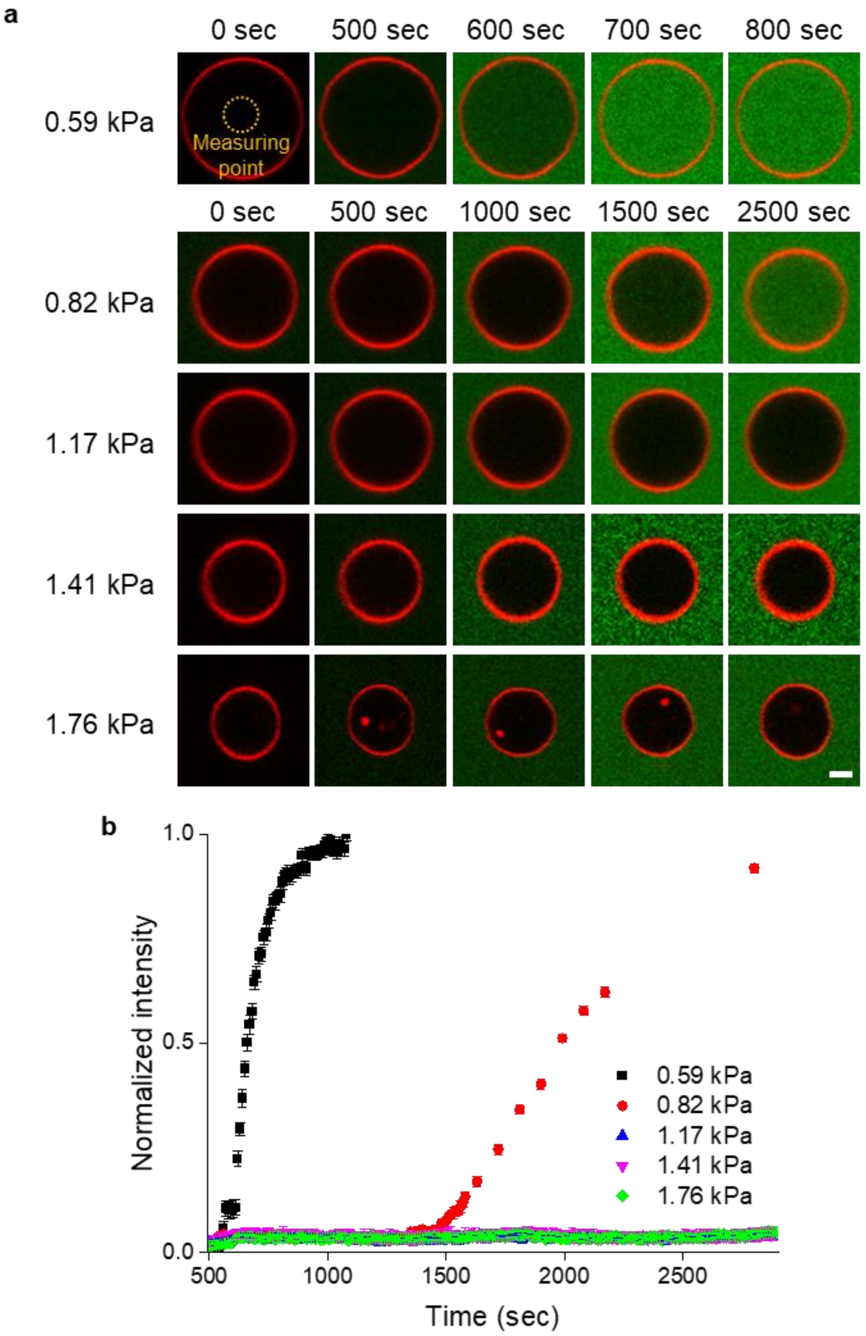

Supplementary Figure 7. (a) Time-sequential fluorescence microscopy images for evaluating the tight sealing of the 3DFLBs. When the 3DFLBs are unstable, Alexa Fluor 488 penetrates inside through gaps. Scale bar: 5 µm. (b) Change in the normalized fluorescence intensity inside the 3DFLBs over time. The slight increase in the normalized intensity at pressures greater than 1.17 kPa represents the background noise associated with the introduction of the fluorescent dye.

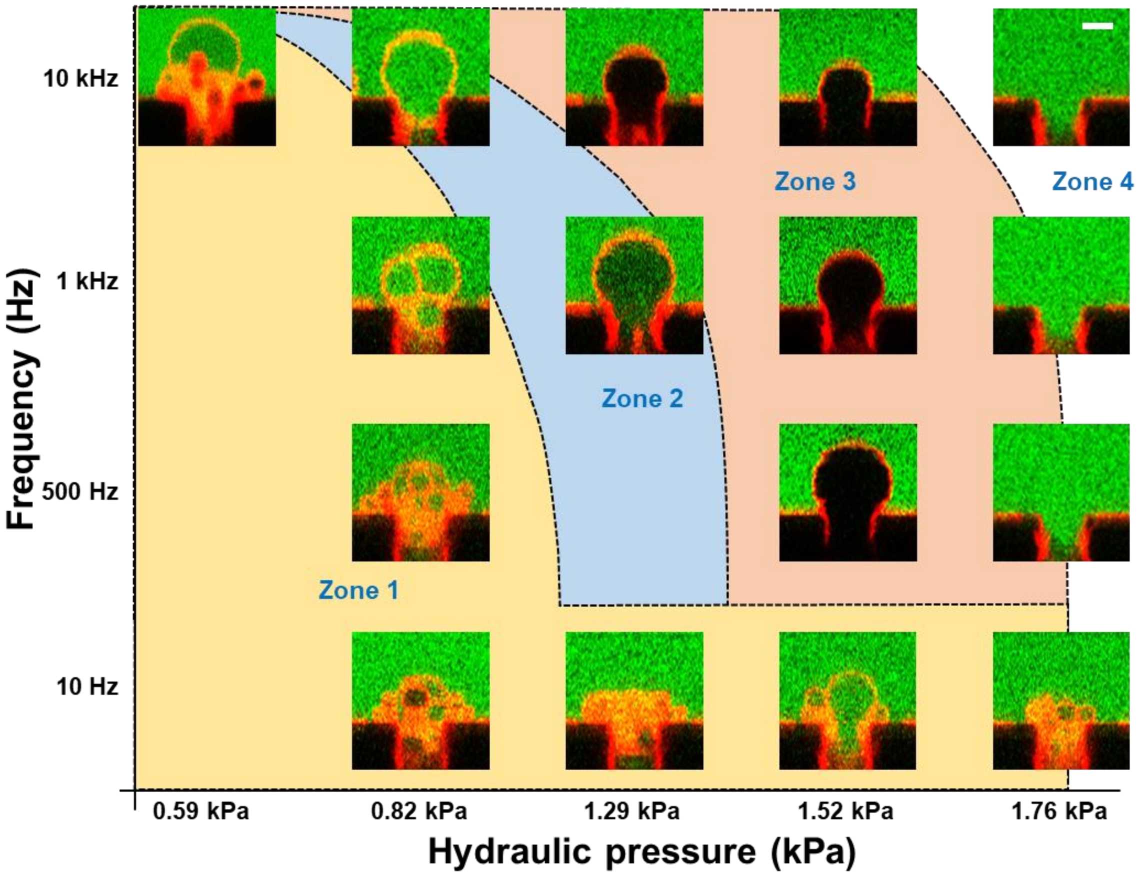

Supplementary Figure 8. Tunable shape and sealing characteristics of 3DFLBs fabricated in KCl solution (physiological ionic condition: 300 mOsml/L) at various AC frequencies and hydraulic pressures. The cross-sectional confocal fluorescence microscopy images were captured at different AC frequencies and hydraulic pressures. Scale bar: 5 μm.

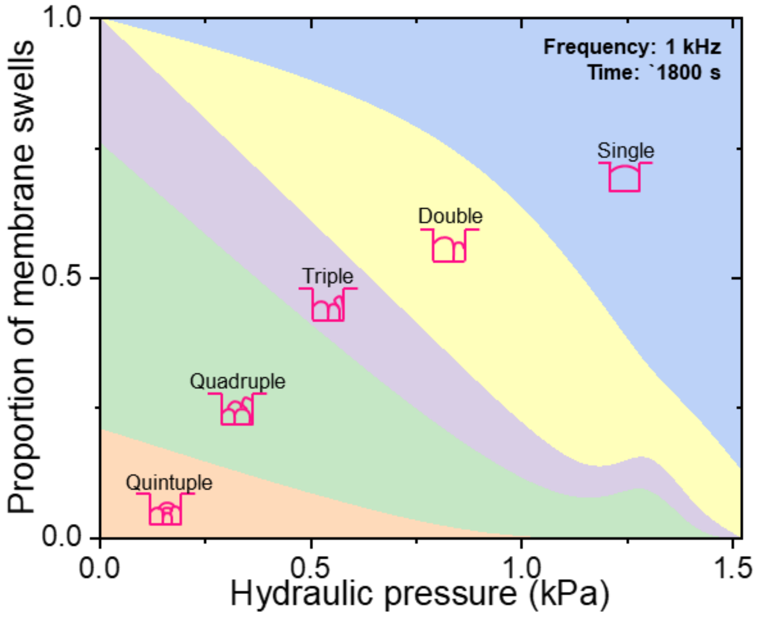

Supplementary Figure 9. Proportion of membrane swells as a function of hydraulic pressure at the end of the early stage (1800 s), for 3DFLBs produced at an AC frequency of 1 kHz in KCl solution (physiological ionic condition; 300 mOsml/L).

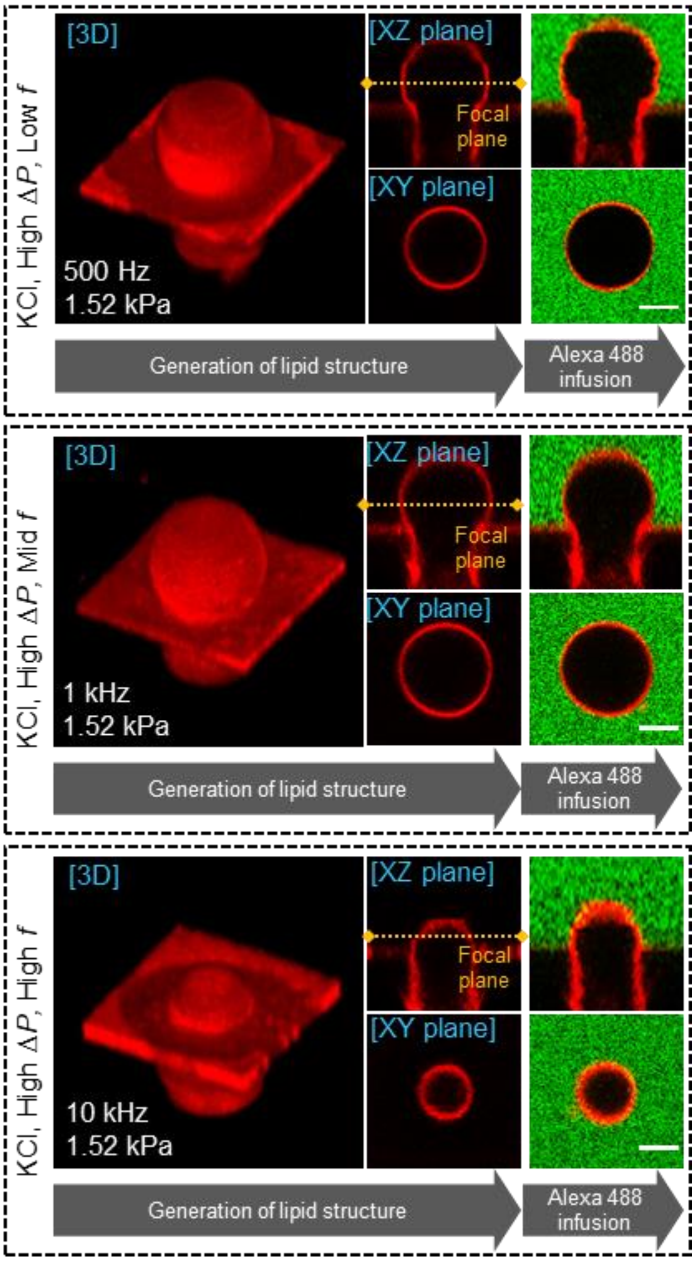

Supplementary Figure 10. 3D reconstructed and cross-sectional confocal fluorescence microscopy images of tightly sealed 3DFLBs formed at frequencies of (top) 500 Hz, (middle) 1 kHz, and (bottom) 10 kHz and 1.52 kPa hydraulic pressure in KCl solution. Scale bar: 5 μm.

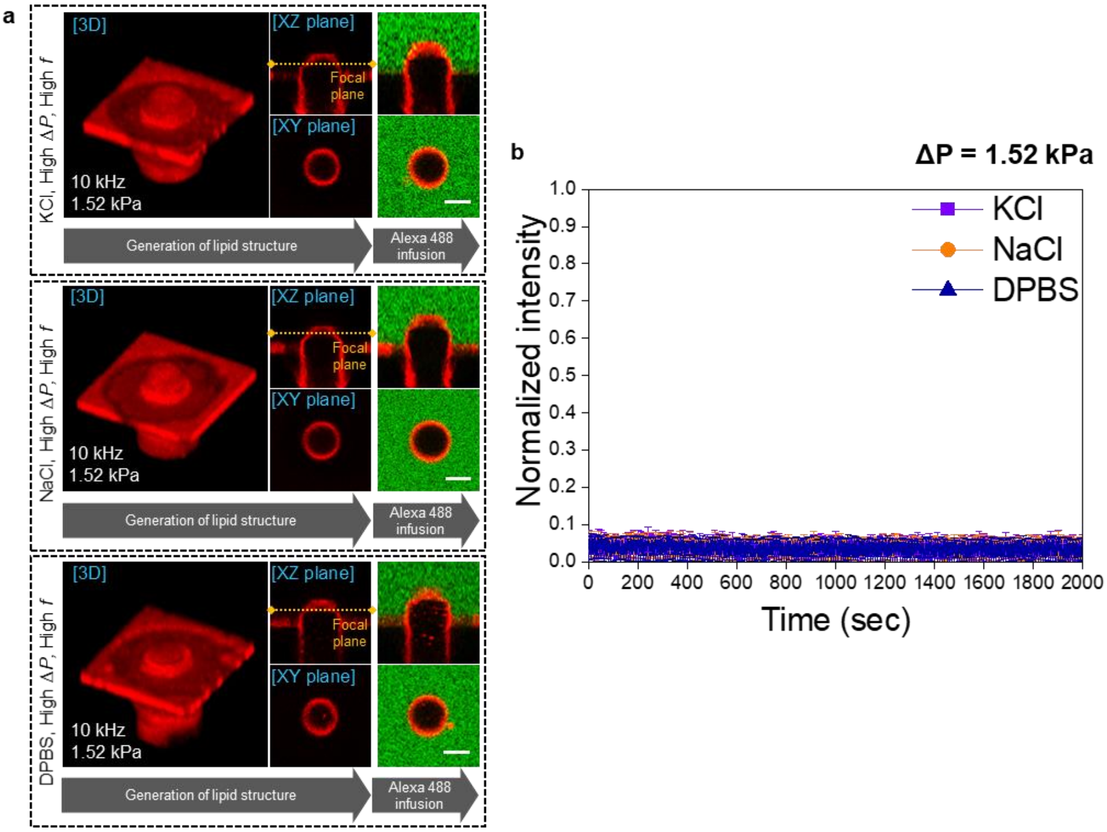

Supplementary Figure 11. (a) 3D reconstructed and cross-sectional confocal fluorescence microscopy images of tightly sealed 3DFLBs generated at an AC frequency of 10 kHz and a hydraulic pressure of 1.52 kPa in (top) KCl solution, (middle) NaCl solution, and (bottom) DPBS solution. All the solutions represent physiologically ionic conditions (300 mOsml/L). Scale bar: 5 μm. (b) Change in the normalized fluorescence intensity inside the 3DFLBs over time. Tightly sealed 3DFLBs are produced at the same pressure of 1.52 kPa in different physiological solutions; therefore, the normalized fluorescence intensity does not change over time.

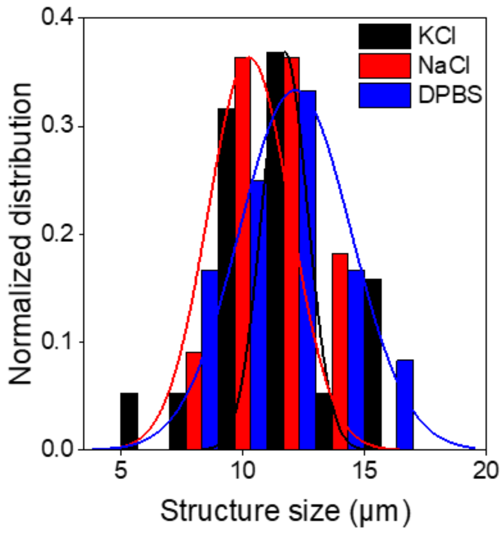

Supplementary Figure 12. Size distributions of the 3DFLBs produced in different buffers (KCl solution, NaCl solution, and DPBS solution). All the solutions were physiologically ionic (300 mOsml/L).

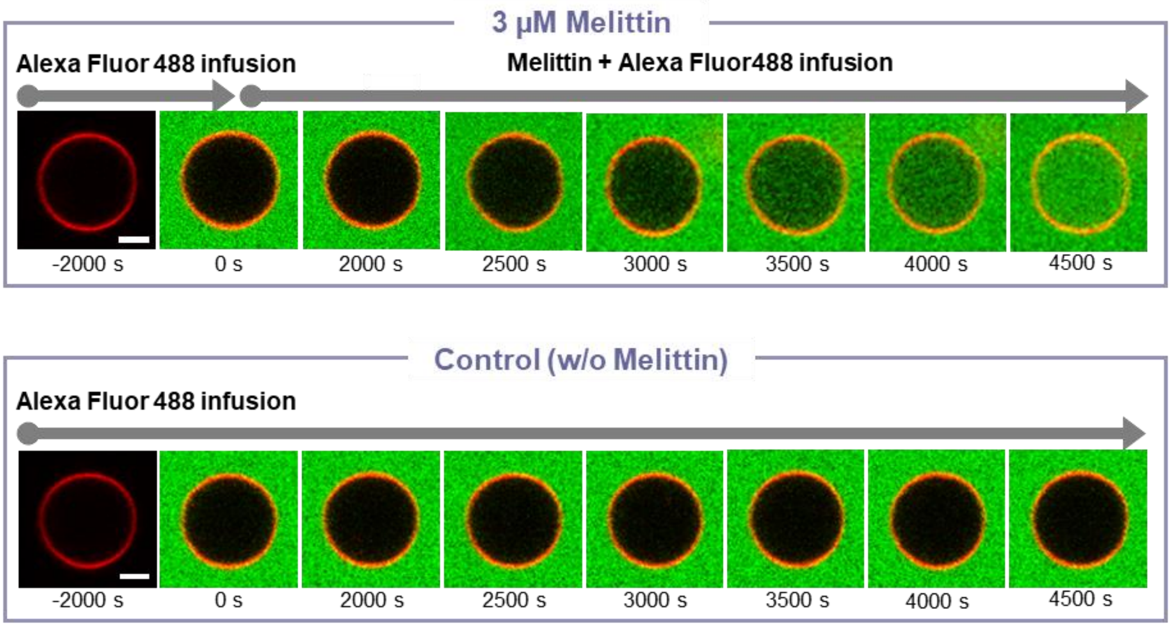

Supplementary Figure 13. Fluorescence microscopy images over time: infusion of Alexa Fluor 488 to confirm the sealing of 3DFLBs and infusion of a mixed solution of Alexa Fluor 488 and melittin to evaluate melittin pore formation and confirm the bilayer membrane structure of the 3DFLBs. For the comparison, without melittin, the solution was infused as a control experiment. Scale bar: 5 μm.

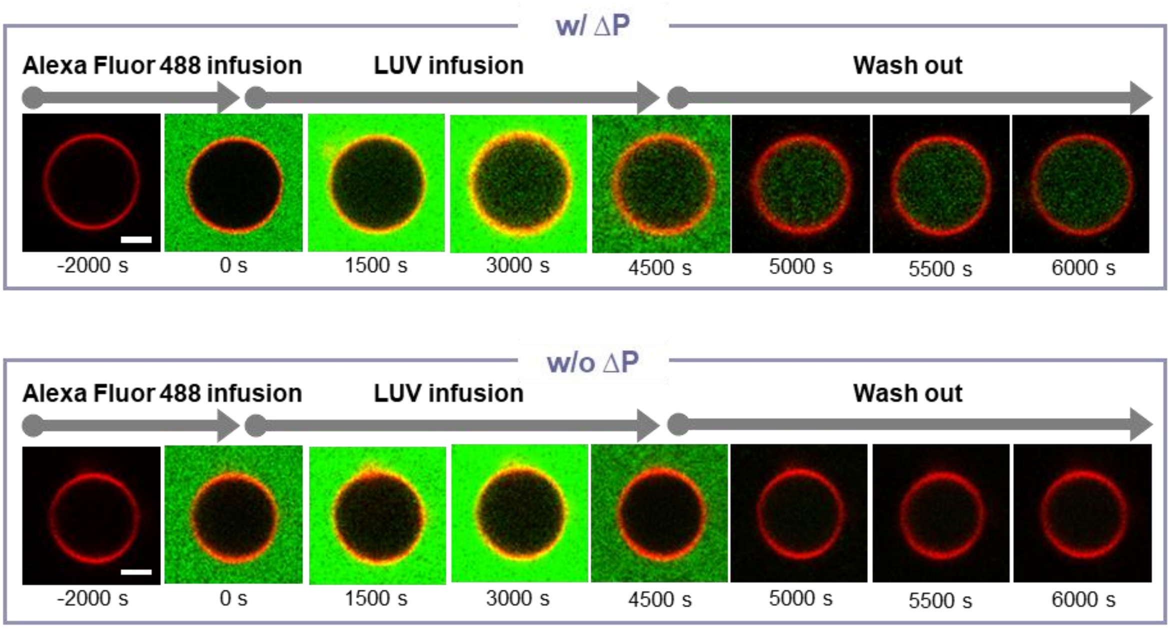

Supplementary Figure 14. Fluorescence microscopy images over time: infusion of Alexa Fluor 488 to confirm the sealing of the 3DFLBs; infusion of LUVs to mimic vesicular transport; and washing out process to evaluate the fusion of LUVs and 3DFLBs by monitoring the fluorescence intensity inside the 3DFLBs. For the comparison, the experiments were conducted in the same manner at the difference of with and without hydraulic pressure. Scale bar: 5 μm.

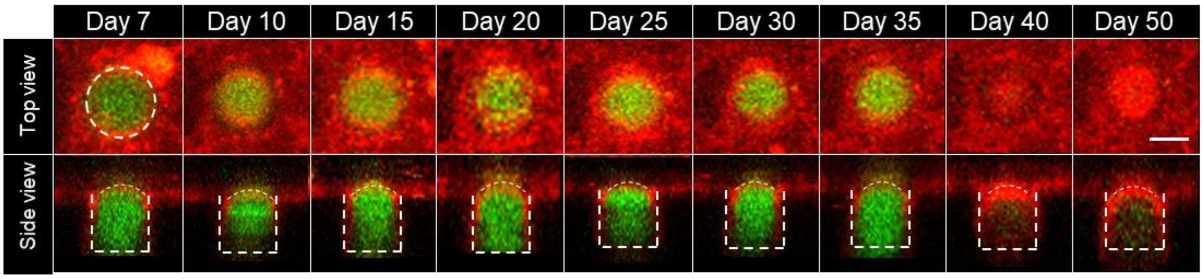

Supplementary Figure 15. Time-sequential cross-sectional confocal fluorescence microscopy images of enclosed 3DFLBs containing Alexa Fluor 488 produced with a hydrogel block (PEGDMA 1000:3400 = 10:5 wt%). At 40 days, Alexa Fluor 488 leaked due to the broken seal of the 3DFLBs. Scale bar: 5 μm.

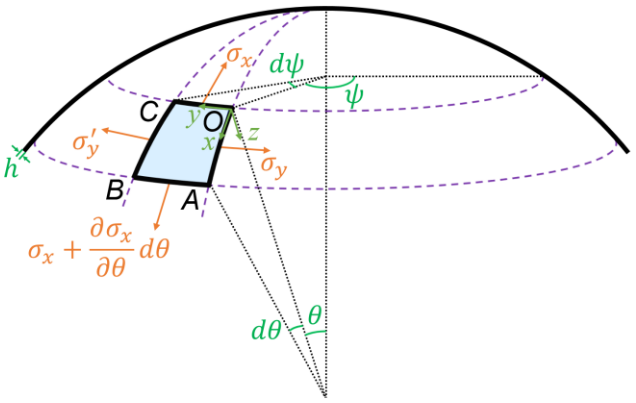

Supplementary Figure 16. A clamped 3DFLBs surface geometry: an infinitesimal volume element of the clamped 3DFLBs surface ($OABC$; blue shaded) is defined by two meridional sections (azimuthal angle $d\psi$ apart) and two conical surfaces (with polar angle difference $d\theta$); h means the thickness of the volume element. σ_x (for face OC) and σ_y (face OA) denote the normal stress (in the unit of N/m^2) regarding the x- and y-axis, respectively. The normal stress on the face AB reads $\sigma_x + \frac{\partial \sigma_x}{\partial \theta} d\theta$, while that of face CB (σ_y') is the same as the face OA (i.e., $\sigma_y = \sigma_y'$) due to the azimuthal symmetry. These infinitesimal elements are integrated over the whole spherical surface.

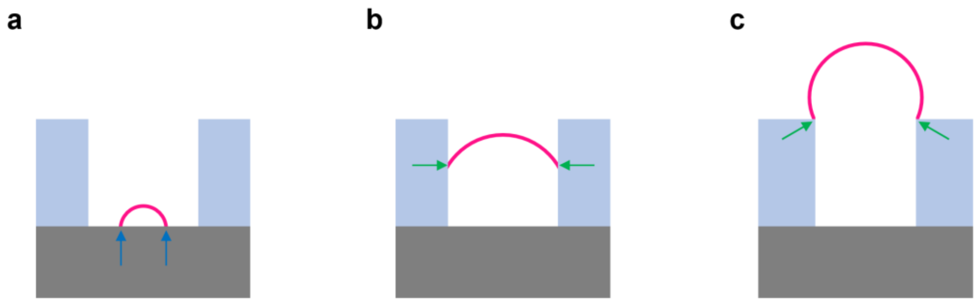

Supplementary Figure 17. Schematic for 2D reduced DOPC-3DFLBs surface models (magenta circular arcs) with structural boundary conditions: (a) *BC-1*: a small bilayer vesicle is on the bottom of the microwell (gray rectangle) along the circular edge (indicated by blue arrows) in no contact with the side wall of the microwell, (b) *BC-2*: a large vesicle is restricted due to a bilayer/sidewall (light blue rectangles) border (green arrows), and (c) *BC-3*: the situation where an outward vesicle is placed on the top of the microwell along its circular edge (green arrows).

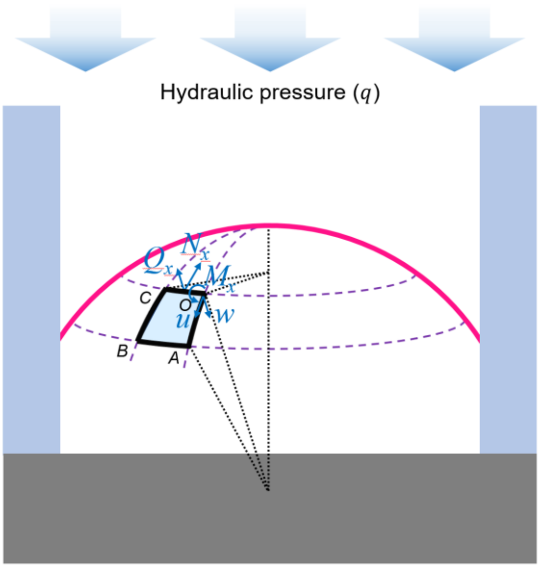

Supplementary Figure 18. Key mechanical descriptors for the 3DFLBs surface geometry. The surface (*OABC*; blue shaded) denotes the infinitesimal volume element of the clamped 3DFLBs surface; the magenta circular arc indicates a 2D reduced 3DFLBs surface. N_x is the force per unit length along the x-axis (see Eq. 17); Q_x is a vertical shearing force acting on the face normal to the x-axis; M_y is the bending moment per unit length, parallel to the y-axis (Eq. 20); u and w respectively denote tangential displacement along the x-axis and the displacement in the direction normal to the spherical surface.

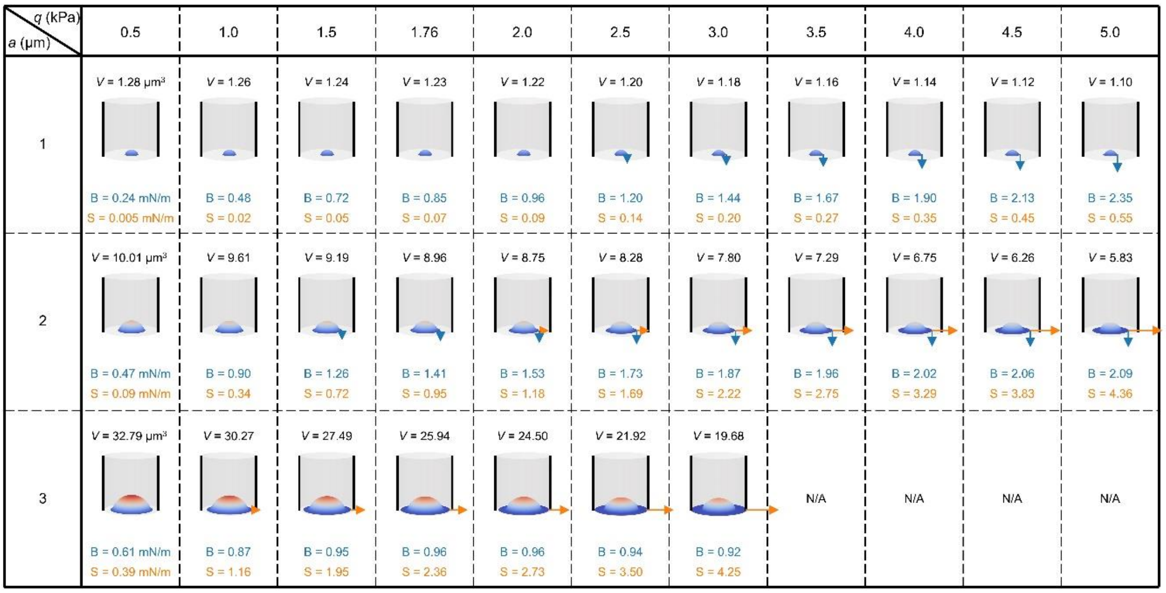

Supplementary Figure 19. Mechanical changes of 3DFLBs upon applying pressure (q) at different initial sphere radii (a) for *BC-1*. Each panel displays the 3D surface plot (color-coded by height), the volume above the bottom of the microwell (color-coded space), and tensions acting at the interface toward the bottom (B; blue arrow) and the side wall (S; orange arrow); those ≤ 1 mN/m are not displayed. The divided tensions S and B respectively denote the horizontal and vertical components of the vector sum of N_x and Q_x. Vertical black solid lines indicate the side wall. For a = 3 μm, the system violates *BC-1* at q = 3.5, 4.0, 4.5, and 5.0 kPa because the 3DFLBs reaches the side wall.

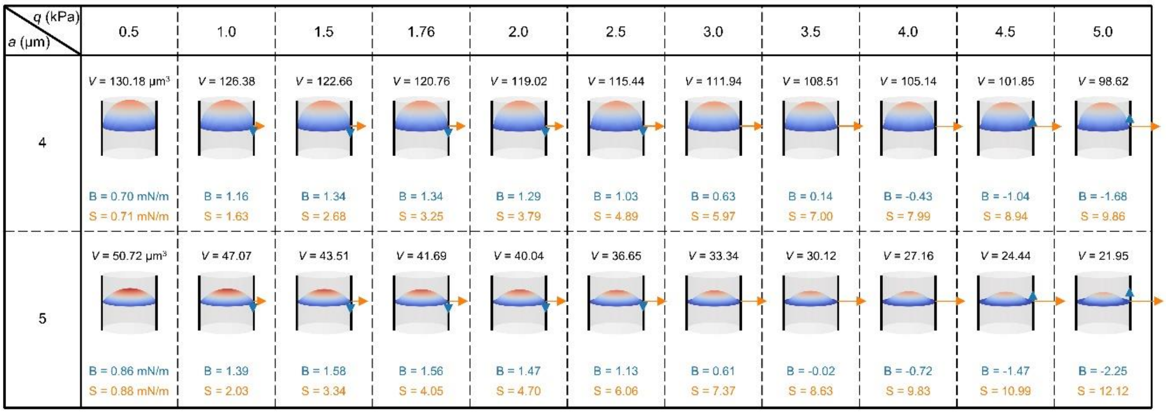

Supplementary Figure 20. Mechanical changes of 3DFLBs upon applying pressure (q) at different initial sphere radii (a) for *BC-2*. Each panel displays the 3D surface plot (color-coded by height), the volume of the color-coded space, and tensions acting at the interface toward the bottom (B; blue arrow) and the side wall (S; orange arrow); those ≤ 1 mN/m are not displayed. The divided tensions S and B respectively denote the horizontal and vertical components of the vector sum of N_x and Q_x. Vertical black solid lines are the side wall.

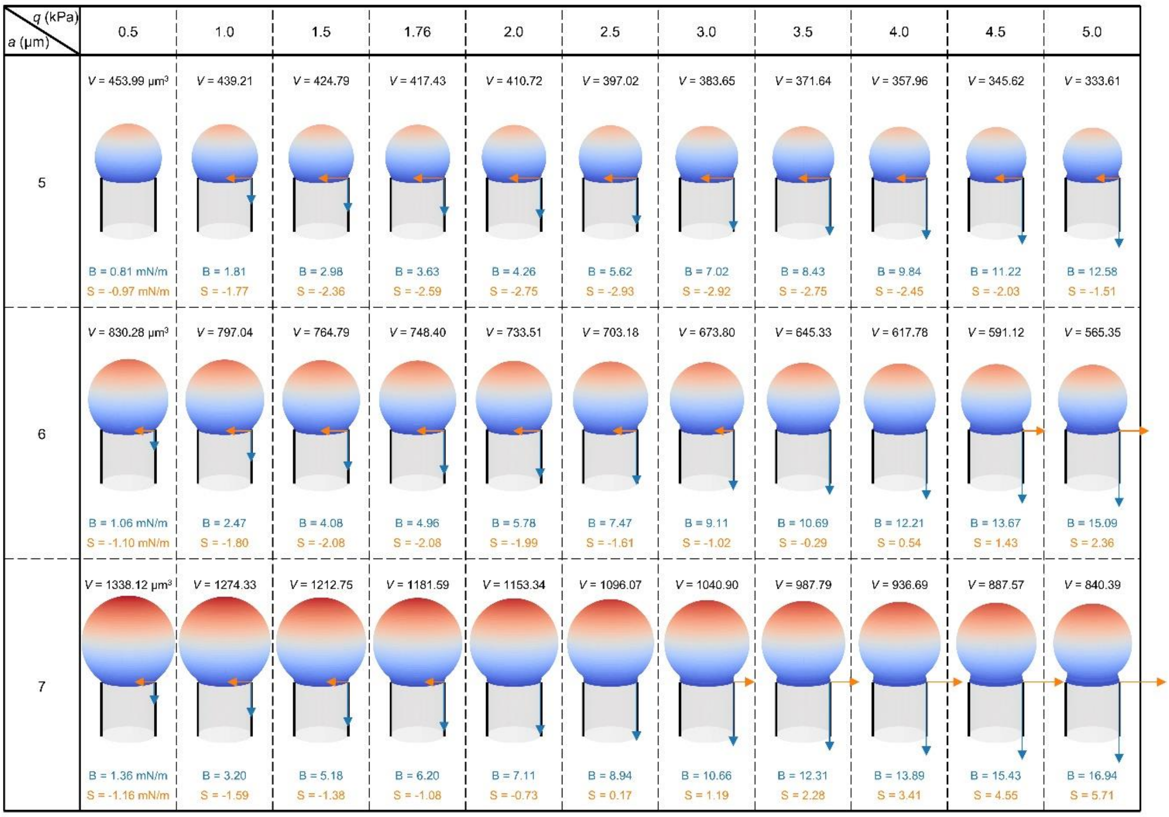

Supplementary Figure 21. Mechanical changes of 3DFLBs upon applying pressure (q) at different initial sphere radii (a) for *BC-3*. Each panel displays the 3D surface plot (color-coded by height), the volume of the color-coded space, and tensions acting at the interface toward the bottom (B; blue arrow) and the side wall (S; orange arrow); those ≤ 1 mN/m are not displayed. The divided tensions S and B respectively denote the horizontal and vertical components of the vector sum of N_x and Q_x. Vertical black solid lines are the side wall.

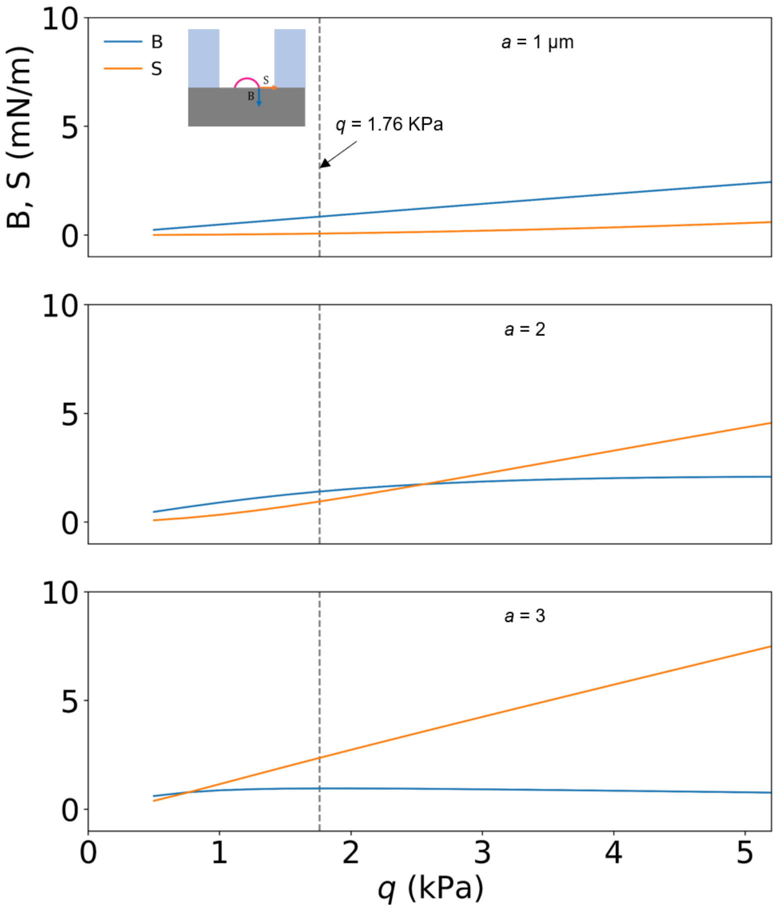

Supplementary Figure 22. Nonlinear changes of the membrane tensions acting toward the bottom (B; blue lines) and the side wall (S; orange lines) for *BC-1* (as shown schematically on the top-left corner). (top) initial sphere radius a = 1; (middle) a = 2; (bottom) a = 3 μm.

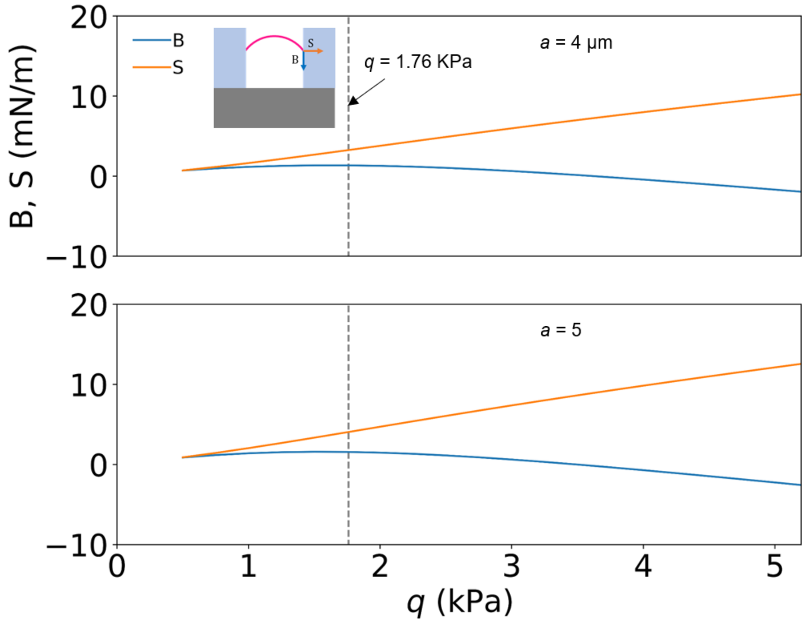

Supplementary Figure 23. Nonlinear changes of the membrane tensions acting toward the bottom (B; blue lines) and the side wall (S; orange lines) for *BC-2* (as shown schematically on the top-left corner). (top) initial sphere radius $a = 4$; (bottom) $a = 5$ µm.

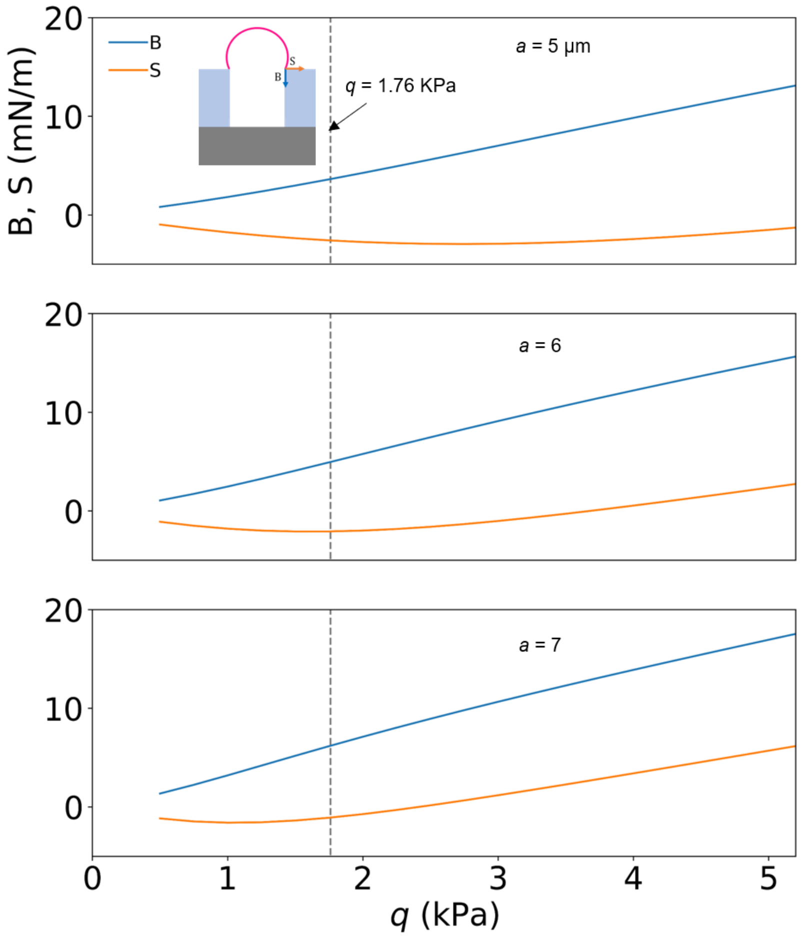

Supplementary Figure 24. Nonlinear changes of the membrane tensions acting toward the bottom (B; blue lines) and the side wall (S; orange lines) for *BC-3* (as shown schematically on the top-left corner). (top) initial sphere radius a = 6; (bottom) a = 7 μm.

[Supplementary Table]

Parameter	Value	Unit	Description
E	1.3×10^{4}	kPa	Young's modulus
v	0.450 - 0.501	(unitless)	Poisson ratio
h	40	Å	Thickness of 3DFLBs
q	0.5 - 10	kPa	Additional hydraulic pressure (Δp)
p_0	102.65	kPa	Default pressure in the microfluidic channel
δ	6.64×10^{-1}	(unitless)	Exponent for the pressure dependence of flexural rigidity
κ	10^{2}	(unitless)	Constant for deformation effect
a	1 - 7	µm	Radius of curvature
b	5.32×10^{4}	(unitless)	Constant for the pressure dependence of flexural rigidity
c	3.28×10^{11}	kPa	Constant for the pressure dependence of flexural rigidity
d	1.81×10^{-5}	(unitless)	Constant for the pressure dependence of flexural rigidity
θ_c^1	75	° (degree)	Polar angle at vesicle/microwell border (*BC-1*)
θ_c^2	53 - 90	°	Polar angle at vesicle/microwell border (*BC-2*)
θ_c^3	138 -145	°	Polar angle at vesicle/microwell border (*BC-3*)

Supplementary Table 1. Young's modulus (E) is chosen from the atomic force microscopy (AFM) experiment on the DOPC bilayer[4]; Poisson ratio (v) of the DOPC bilayer is estimated as 0.450–0.501 according to slightly different experimental conditions[5]; the 3DFLBs thickness is set to 40 Å following the measurement on DOPC-GUV (giant unilamellar vesicle)[6]. Polar angles are adjusted for each of the boundary conditions (*BC-1 – BC-3*).

[Supplementary References]

1. Timoshenko, S. P. & Gere, J. M. *Theory of Elastic Stability*. *Courier Corporation* (McGraw-Hill Book Company, 1985).

2. Love, A. E. H. *A Treatise on the Mathematical Theory of Elasticity*. *Cambridge University Press* (Cambridge University Press, 2013).

3. Tu, Z. C. & Ou-Yang, Z. C. Elastic Theory of Low-Dimensional Continua and Its Applications in Bio- and Nano-Structures. *J Comput Theor Nanosci* **5**, 422–448 (2008).

4. Et-Thakafy, O. *et al.* Mechanical Properties of Membranes Composed of Gel-Phase or Fluid-Phase Phospholipids Probed on Liposomes by Atomic Force Spectroscopy. *Langmuir* **33**, 5117–5126 (2017).

5. Terzi, M. M., Deserno, M. & Nagle, J. F. Mechanical properties of lipid bilayers: a note on the Poisson ratio. *Soft Matter* **15**, 9085–9092 (2019).

6. McPhee, C. I., Zoriniants, G., Langbein, W. & Borri, P. Measuring the Lamellarity of Giant Lipid Vesicles with Differential Interference Contrast Microscopy. *Biophys J* **105**, 1414–1420 (2013).

7. Purushothaman, S., Cicuta, P., Ces, O. & Brooks, N. J. Influence of High Pressure on the Bending Rigidity of Model Membranes. *J Phys Chem B* **119**, 9805–9810 (2015).

8. Kierzenka, J. & Shampine, L. F. A BVP solver based on residual control and the Maltab PSE. *ACM Transactions on Mathematical Software* **27**, 299–316 (2001).

9. Ascher, U. M., Mattheij, R. M. M. & Russell, R. D. *Numerical solution of boundary value problems for ordinary differential equations*. (SIAM, 1995).

10. Virtanen, P. *et al.* SciPy 1.0: fundamental algorithms for scientific computing in Python. *Nat Methods* **17**, 261–272 (2020).
