# Supplementary material for: Control of artificial membrane fusion in physiological ionic solutions beyond the limits of electroformation
Source: Nat Commun. 2024 May 28;15:4524. doi: 10.1038/s41467-024-48875-0 (PMC11133453; doi:10.1038/s41467-024-48875-0)
Supplement: Supplementary file 3 — Description of Additional Supplementary Files [file 41467_2024_48875_MOESM3_ESM.pdf]

Description of Additional Supplementary Files

File name: Supplementary Movie 1
Description: Time-lapse observation of explosive membrane swells at an AC frequency of 1 kHz during conventional electroformation without hydraulic pressure for 1800 s from the beginning of rehydration using confocal fluorescence microscopy in the X-Y plane. The dried lipid stack patterned in the microwell develops into multiple multilayer membrane swells after encountering sucrose solution under the AC electric field. During this sequence, the multiple membrane swells progressively fuse with each other; however, this process is relatively slow compared to that observed during hydraulic pressure-assisted electroformation.

File name: Supplementary Movie 2
Description: Time-lapse observation of a single lipid membrane swell growing at an AC frequency of 1 kHz and a hydraulic pressure of 1.76 kPa for 1800 s from the beginning of rehydration using confocal fluorescence microscopy in the X-Y plane. The applied hydraulic immediately causes the membrane swells to fuse and form a single multilayer lipid structure. We can observe this quick process only under the focal area of confocal fluorescence microscopy, causing the initial observation to be missed due to its location outside the focal plane. As the single multilayer lipid structure grows, the lipid membrane passes through the focal region, and a ring-shaped single bilayer is momentarily observed.
